# Inhibition of angiogenesis by leflunomide via targeting the soluble ephrin-A1/EphA2 system in bladder cancer

**DOI:** 10.1038/s41598-018-19788-y

**Published:** 2018-01-24

**Authors:** Maolin Chu, Chunying Zhang

**Affiliations:** Department of Urology, The Second Affiliated Hospital, Harbin Medical University, 246 Xuefu St., Nan Gang District, Harbin, China

## Abstract

Angiogenesis plays an important role in bladder cancer (BCa). The immunosuppressive drug leflunomide has attracted worldwide attention. However, the effects of leflunomide on angiogenesis in cancer remain unclear. Here, we report the increased expression of soluble ephrin-A1 (sEphrin-A1) in supernatants of BCa cell lines (RT4, T24, and TCCSUP) co-cultured with human umbilical vein endothelial cells (HUVECs) compared with that in immortalized uroepithelial cells (SV-HUC-1) co-cultured with HUVECs. sEphrin-A1 is released from BCa cells as a monomeric protein that is a functional form of the ligand. The co-culture supernatants containing sEphrin-A1 caused the internalization and down-regulation of EphA2 on endothelial cells and dramatic functional activation of HUVECs. This sEphrin-A1/EphA2 system is mainly functional in regulating angiogenesis in BCa tissue. We showed that leflunomide (LEF) inhibited angiogenesis in a N-butyl-N-(4-hydroxybutyl)-nitrosamine (BBN)-induced bladder carcinogenesis model and a tumor xenograft model, as well as in BCa cell and HUVEC co-culture systems, via significant inhibition of the sEphrin-A1/EphA2 system. Ephrin-A1 overexpression could partially reverse LEF-induced suppression of angiogenesis and subsequent tumor growth inhibition. Thus, LEF has a significant anti-angiogenesis effect on BCa cells and BCa tissue via its inhibition of the functional angiogenic sEphrin-A1/EphA2 system and may have potential for treating BCa beyond immunosuppressive therapy.

## Introduction

Bladder cancer (BCa) is the most common urinary tract cancer with a high recurrence rate after transurethral resection. The heterogeneity of BCa patients leads to the poor responses of many patients to traditional chemotherapy regimens, which are also less effective on invasive or higher-grade tumors^1^. Angiogenesis is a critical step in the progression of BCa^2^, and therefore, effective antiangiogenic therapy should be optimized and might require interference with multiple angiogenic pathways.

Ephrins and their Eph receptors have been identified as critical regulators of angiogenesis^3^. The ephrins comprise a family of ligands for Eph receptor tyrosine kinases that have been characterized as glycosyl phosphatidyl inositol (GPI)-anchored (ephrinA) or transmembrane (ephrinB) cell surface proteins^4^. Ephrin-A1, the first identified ligand for an Eph receptor, is overexpressed in BCa^5^ and induces endothelial cell migration and capillary assembly *in vitro*^6^, suggesting a critical role in inducing tumor angiogenesis through its EphA2 receptor localized primarily on tumor-associated vascular endothelial cells^7,8^. Membrane-anchored ephrin-A1 is widely considered the endogenous functional form of the ligand^9^; hence, the important physiological functions of ephrin-A1 are largely dependent on cell–cell contact^10^. However, based on a recent study showing that ephrin-A1 may be released from some types of tumor cells and can elicit cellular responses that are not dependent on juxtacrine interactions^11^, we became interested in determining whether soluble ephrin-A1 (sEphrin-A1) is secreted by BCa cells and thereafter exerts a more extensive pro-angiogenic effect on endothelial cells through a paracrine pathway in complex tumors, such as BCa.

Leflunomide (LEF), an immunosuppressive agent effective in the control of rheumatoid arthritis, is distinguished as an agent uniquely capable of inhibiting angiogenesis^12,13^ and subsequently exhibiting great antitumor potential^14^. However, the anti-angiogenesis molecular mechanisms, as well as the therapeutic effects, of LEF on BCa remain unknown. Therefore, we have quantitatively analyzed and identified the pro-angiogenesis sEphrin-A1/EphA2 system in BCa tissue and subsequently investigated the effect of LEF on BCa cells, microvessel sprouting in aortic ring *ex vivo* assays, the N-butyl-N-(4-hydroxybutyl)-nitrosamine (BBN)-induced BCa mouse model and a tumor xenograft model *in vivo* to explore the anti-angiogenesis molecular mechanisms of LEF. Specifically, we determined whether LEF has antitumor ability through the inhibition of sEphrin-A1/EphA2 system-mediated angiogenesis.

## Results

### Increased expression of sEphrin-A1 from BCa cells down-regulates EphA2 expression on HUVECs

**Figure 1:**
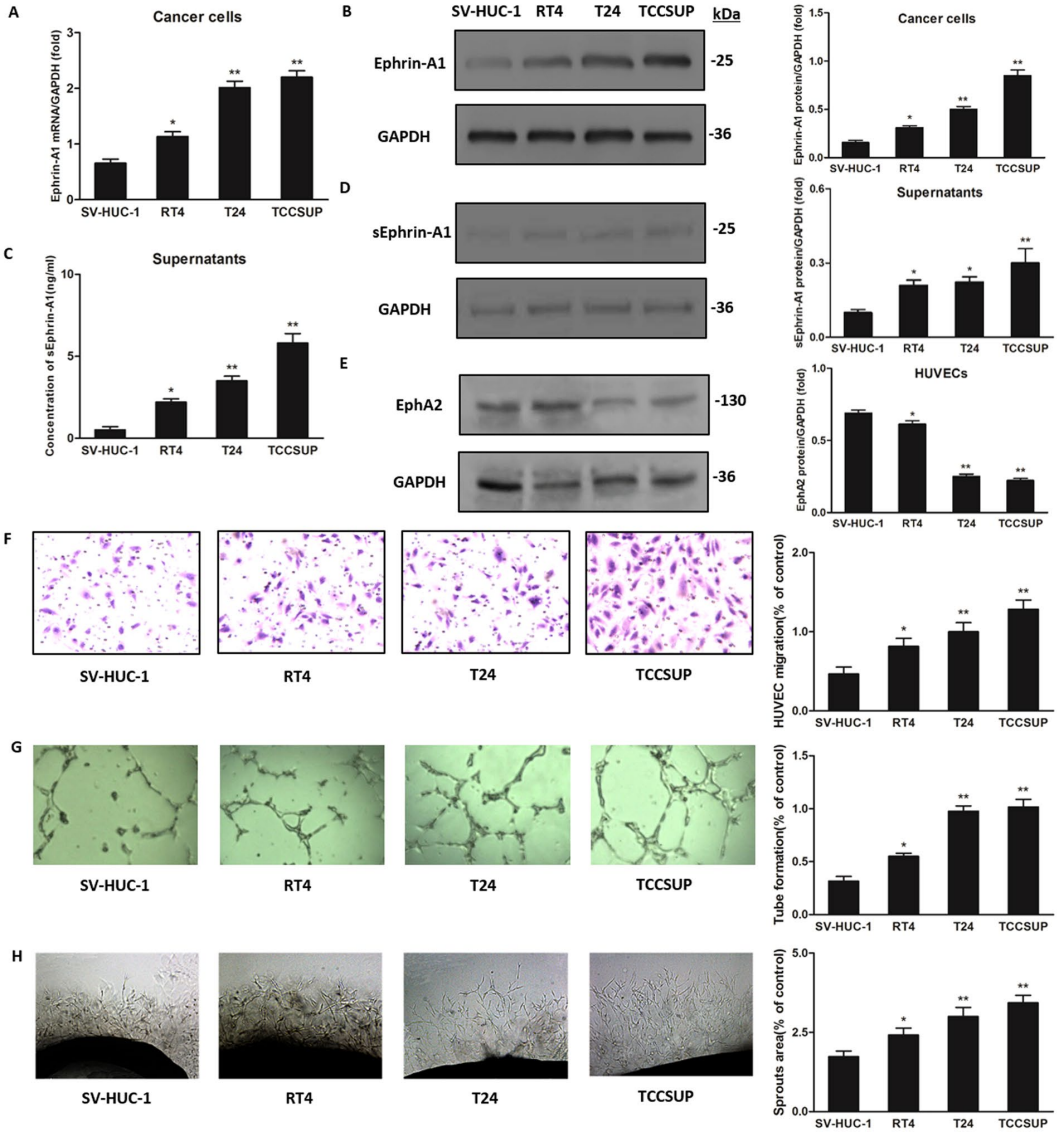
Increased expression of ephrin-A1 in BCa cells and HUVECs co-cultures compared to SV-HUC-1 cells and HUVECs co-culture. BCa cells (RT4, T24, TCCSUP cells) were co-cultured with HUVECs for 48 h, respectively. (**A,B**) Real-time PCR (A; n = 3) and western blot analysis (B; n = 3) respectively showed significant up-regulation of ephrin-A1 mRNA and protein expression in BCa cells (n = 3, respectively) co-cultured compared to that in SV-HUC-1 cells (n = 3; *p < 0.05, **p < 0.01). (**C,D**) ELISA (C; n = 3) showed significantly enhanced expression of sEphrin-A1 in the supernatants of BCa cells and HUVECs co-cultures (n = 3, respectively) compared to those from SV-HUC-1 and HUVECs co-culture (n = 3; *p < 0.05, **p < 0.01), which was further verified by western blot assay (D; n = 3, respectively). The increase in ephrin-A1 expression was highest in TCCSUP cells, followed by T24 cells and RT4 cells. (**E**) Western blot analysis demonstrated significant down-regulation of EphA2 protein expression in HUVECs from the lower chambers of BCa cell and HUVEC co-cultures (n = 3) compared to that in HUVECs from SV-HUC-1 and HUVEC co-cultures (n = 3; *p < 0.05, **p < 0.01). The changing trends of EphA2 protein expression in HUVECs were contrary to ephrin-A1 expression in BCa cells; down-regulation was lowest in HUVECs co-cultured with TCCSUP cells, slightly higher in those co-cultured with T24 cells and highest in those co-cultured with RT4 cells. (**F,G**) Transwell assay (F; n = 3) and tube formation test (G; n = 3) demonstrated significant up-regulation in migration and capillary-like structure formation of HUVECs respectively under treatment of supernatants from BCa cells and HUVECs co-cultures (n = 3, respectively) compared to that from SV-HUC-1 cells and HUVECs co-culture (n = 3; *p < 0.05, **p < 0.01). (**H**) *Ex vo* aortic ring angiogenesis assay showed similar changes in transwell assays and tube formation tests (n = 3, respectively; *p < 0.05, **p < 0.01). The results of vascular functional experiments demonstrated that vascularity was highest under treatment of supernatants from TCCSUP cell and HUVEC co-cultures, moderate in those from T24 cell and HUVEC co-cultures, and lowest in those from RT4 cell and HUVEC co-cultures, consistent with the up-regulation of sEphrin-A1 expression in supernatants. Original magnification = ×5. Results are expressed as the mean ± S.E.M.

We first determined the expression of ephrin-A1 in human BCa cell lines (RT4, T24, and TCCSUP) compared with immortalized uroepithelial cells (SV-HUC-1) using a BCa cell and HUVEC co-culture system. Real-time PCR and western blotting showed significantly increased mRNA and protein expression of ephrin-A1 in co-cultured BCa cells compared to that in SV-HUC-1 cells (*p* < 0.05, Fig. 1A,B). ELISA showed significantly enhanced expression of sEphrin-A1 in the supernatants of BCa cells and HUVECs co-cultures compared to those from SV-HUC-1 and HUVECs co-culture (*p* < 0.05, Fig. 1C), which was further verified by western blot assay (*p* < 0.05, Fig. 1D). The increase in ephrin-A1 expression was highest in TCCSUP cells, followed by T24 cells and RT4 cells. Next, we observed the significant down-regulation of EphA2 protein expression in HUVECs from the lower chambers of BCa cell and HUVEC co-cultures compared to that in HUVECs from SV-HUC-1 and HUVEC co-cultures. Interestingly, the changing trends in EphA2 protein expression in HUVECs were contrary to ephrin-A1 expression in BCa cells; down-regulation was lowest in HUVECs co-cultured with TCCSUP cells, slightly higher in those co-cultured with T24 cells and highest in those co-cultured with RT4 cells (*p* < 0.05, Fig. 1E).

### BCa cell and HUVEC co-culture supernatants increased HUVEC migration, tube formation *in vitro* and microvessel sprouting *ex vivo*

We examined whether the increased expression of sEphrin-A1 after secretion into co-culture supernatants would promote HUVEC migration and tube formation by using transwell analysis and tube formation tests *in vitro*. The migration of HUVECs induced by the supernatants from co-cultured BCa cells and HUVECs was significantly greater than that with supernatants from SV-HUC-1 and HUVEC co-cultures (*p* < 0.05, Fig. 1F). Furthermore, we observed significantly enhanced tube formation, as determined by microvessel counts in Matrigel, in supernatants from co-cultured BCa cells and HUVECs compared with those from SV-HUC-1 and HUVEC co-cultures (*p* < 0.05, Fig. 1G). Supernatants from co-cultures were also used to perform mouse aortic ring assays *ex vivo*, and we observed similar changes in transwell assays and tube formation tests (*p* < 0.05, Fig. 1H). Interestingly, the results of vascular functional experiments demonstrated that vascularity was highest under treatment of supernatants from TCCSUP cell and HUVEC co-cultures, moderate in those from T24 cell and HUVEC co-cultures, and lowest in those from RT4 cell and HUVEC co-cultures, consistent with the up-regulation of sEphrin-A1 expression in supernatants (*p* < 0.05, Fig. 1F–H).

### Ephrin-A1 stimulation, overexpression and knockdown

**Figure 2:**
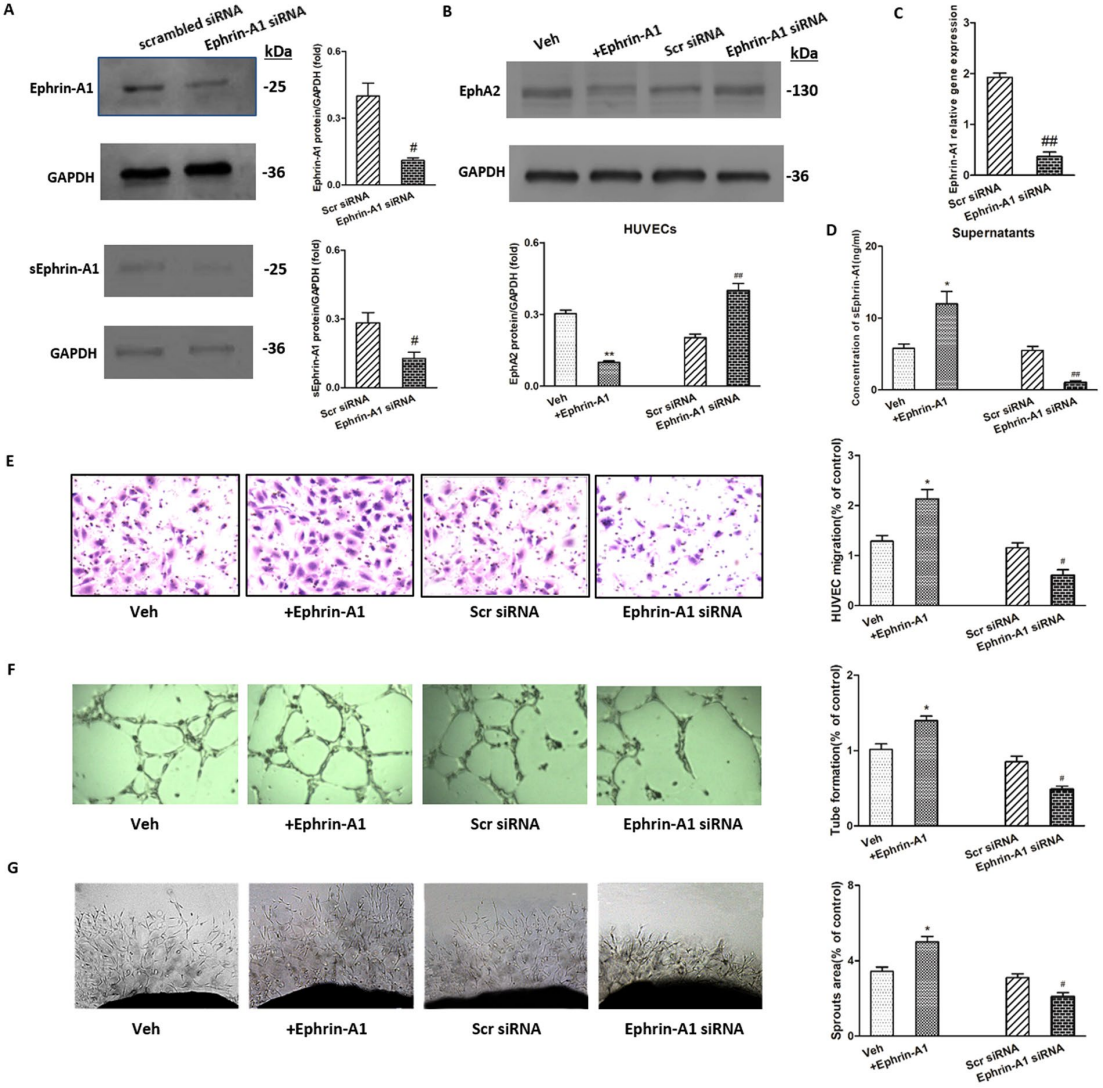
The modulation of ephrin-A1 and EphA2 expression alongside interventions of ephrin-A1 stimulation or knockdown. (**A**,**C**) Significant decrease in the expression of ephrin-A1 were observed at both protein (**A**) and mRNA (**C**) levels in ephrin-A1 siRNA-treated TCCSUP cells compared to control siRNA-treated cells (n = 3, respectively; ^#^*p* < 0.05, ^##^*p* < 0.01 versus Scr siRNA). sEphrin-A1 protein expression (A; n = 3) in the co-culture supernatants was significantly down-regulated after ephrin-A1 knockdown (^#^*p* < 0.05 versus Scr siRNA). Three sets of siRNAs for ephrin-A1 gene have been initially checked and the results from most efficient ones are presented. (**B**) EphA2 protein expression in HUVECs co-cultured were analyzed by western blot analysis after ephrin-A1 stimulation/silencing. EphA2 expression in co-cultured HUVECs was significantly down-regulated after ephrin-A1 stimulation (**p < 0.01 versus Veh) and significantly up-regulated after ephrin-A1 knockdown (^##^*p* < 0.01 versus Scr siRNA). (**D**) sEphrin-A1 protein expression in the supernatants of co-cultures were also determined by ELISA. Stimulation/silencing of ephrin-A1 in TCCSUP cells resulted in significant up-regulation/ down-regulation of sEphrin-A1 protein expression in the supernatants (n = 3, respectively; *p < 0.05 versus Veh, ^##^*p* < 0.01 versus Scr siRNA). (**E–G**) Transwell assay (E; n = 3), tube formation test (F; n = 3) and *ex vo* aortic ring angiogenesis assay (G; n = 3) respectively showed significant up-regulation/down-regulation in migration, capillary-like structure formation of HUVECs, and microvessel sprouting under treatment of supernatants from ephrin-A1 stimulation/silencing TCCSUP cells and HUVECs co-cultures (n = 3, respectively; *p < 0.05 versus Veh, ^#^*p* < 0.05 versus Scr siRNA). Original magnification = ×5. Results are expressed as the mean ± S.E.M. Veh = vehicle control. Scr siRNA = scramble siRNA.

Membrane-anchored and soluble forms of ephrin-A1 proteins were up-regulated in BCa cells and HUVECs co-cultures. To further clarify the activity of soluble ephrin-A1, experiments with ephrin-A1 stimulation and overexpression were carefully performed, followed by knockdown assays. Moreover, the TCCSUP cell line was applied for this further evaluation of the biological activity of sEphrin-A1 in the supernatants due to the mostly significant elevation of ephrin-A1 expression. The efficiency of ephrin-A1 overexpression and knockdown was determined by real-time PCR and western blot analysis. After transfection, 150% overexpression and 75% knockdown efficiency were achieved, as shown by both gene and protein expression analyses (Supplementary Fig. 1A,B, Fig. 2A,C). After stimulation, overexpression or knockdown, TCCSUP cells were further co-cultured with HUVECs. The results showed that sEphrin-A1 protein expression in the co-culture supernatants was significantly up-regulated after ephrin-A1 stimulation (*p* < 0.05, Fig. 2D) and overexpression (*p* < 0.05, Supplementary Fig. 1C) and significantly down-regulated after ephrin-A1 knockdown (*p* < 0.01, Fig. 2D). EphA2 expression in co-cultured HUVECs was significantly down-regulated after ephrin-A1 stimulation (*p* < 0.05, Fig. 2B) and significantly up-regulated after ephrin-A1 knockdown (*p* < 0.05, Fig. 2B). Changing trends in HUVEC migration, tube formation and microvessel sprouting induced by co-cultured supernatants were similar to the results of sEphrin-A1 expression (*p* < 0.05, Fig. 2E–G).

### Different bladder cell lines have different sensitivity to LEF *in vitro*

**Figure 3:**
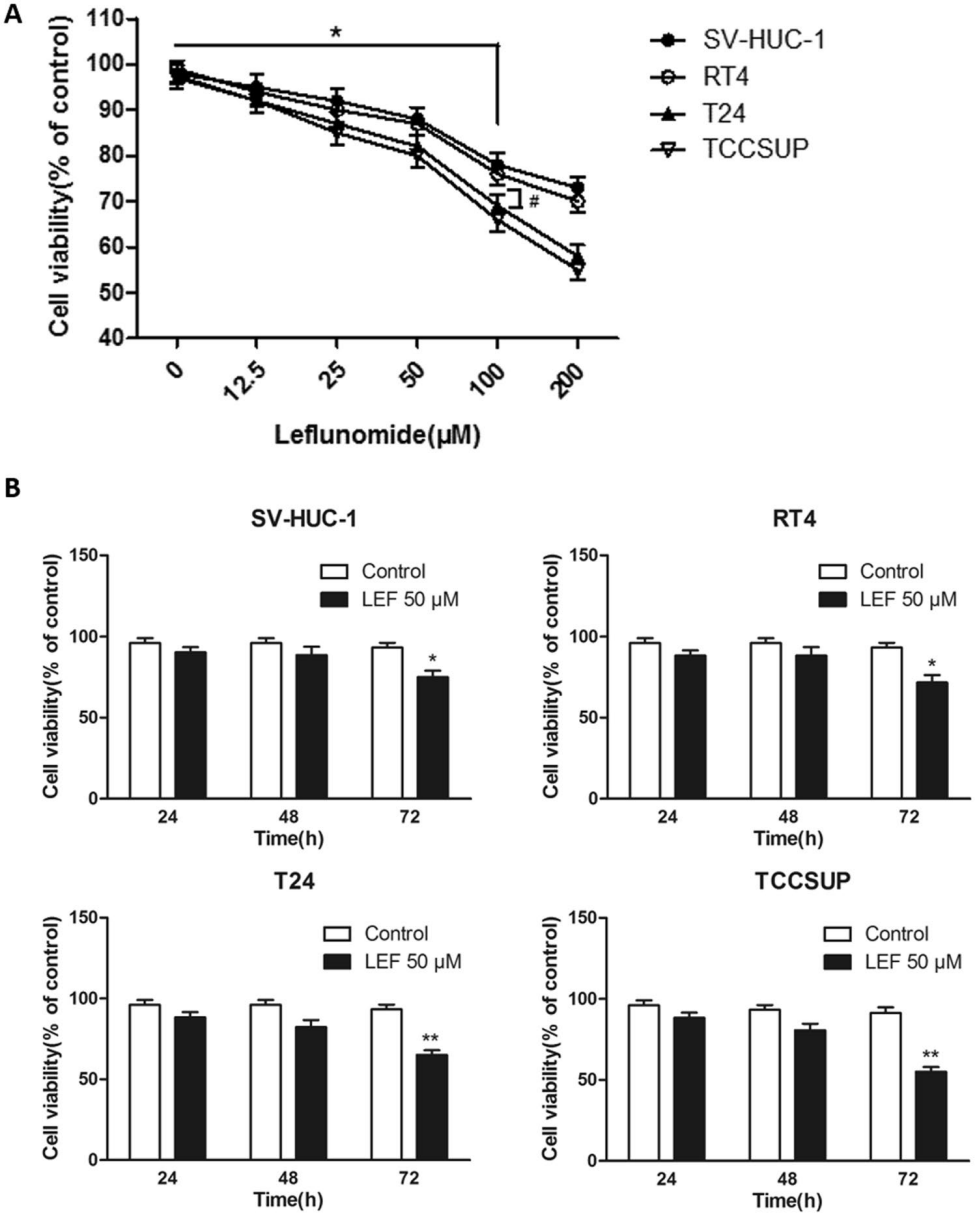
Different bladder cell line has different sensitivity to LEF *in vitro*. (**A**) LEF decreased viability of bladder cells in a dose-dependent manner. Treatment with doses of more than 50 μM LEF, i.e., 100 and 200 μM, significantly reduced the viability of SV-HUC-1 cells by 48 hours (n = 3; *p < 0.05 versus LEF at 0 μM for 0 h), while the viability of T24 and TCCSUP cells exposed to 100 μM LEF for 48 hours was significantly reduced compared to that of SV-HUC-1 cells (n = 3; ^#^*p* < 0.05 versus SV-HUC-1 cells). (**B**) Treatment with LEF at 50 μM for 72 hours significantly reduced the viability of all four cell lines (n = 3, respectively) compared to control (n = 3; *p < 0.05 versus LEF at 0 μM for 0 h). Results are expressed as the mean ± S.E.M.

The viability of immortalized uroepithelial cells (SVHUC-1) and of low-grade (RT4) and high-grade (T24 and TCCSUP) BCa cells treated with 0 to 200 μM LEF for 24, 48, and 72 hours was evaluated. The results showed that LEF reduced the viability of bladder cells in dose- and time-dependent manners. As shown in Fig. 3B, treatment with LEF at 50 μM for 72 hours significantly reduced the viability of all four cell lines compared to control. Treatment with doses of more than 50 μM LEF, i.e., 100 and 200 μM, significantly reduced the viability of SV-HUC-1 cells by 48 hours (*p* < 0.05, Fig. 3A), while the viability of T24 and TCCSUP cells exposed to 100 μM LEF for 48 hours was significantly reduced compared to that of SV-HUC-1 cells (*p* < 0.05, Fig. 3A). Therefore, we selected the 50 μM concentration and the 48 h time point, which do not induce significant cell toxicity, as the following experimental condition to study the influence of LEF on neovascularization in BCa.

### LEF treatment inhibits the up-regulated expression of ephrin-A1 in BCa cell and HUVEC co-culture systems

**Figure 4:**
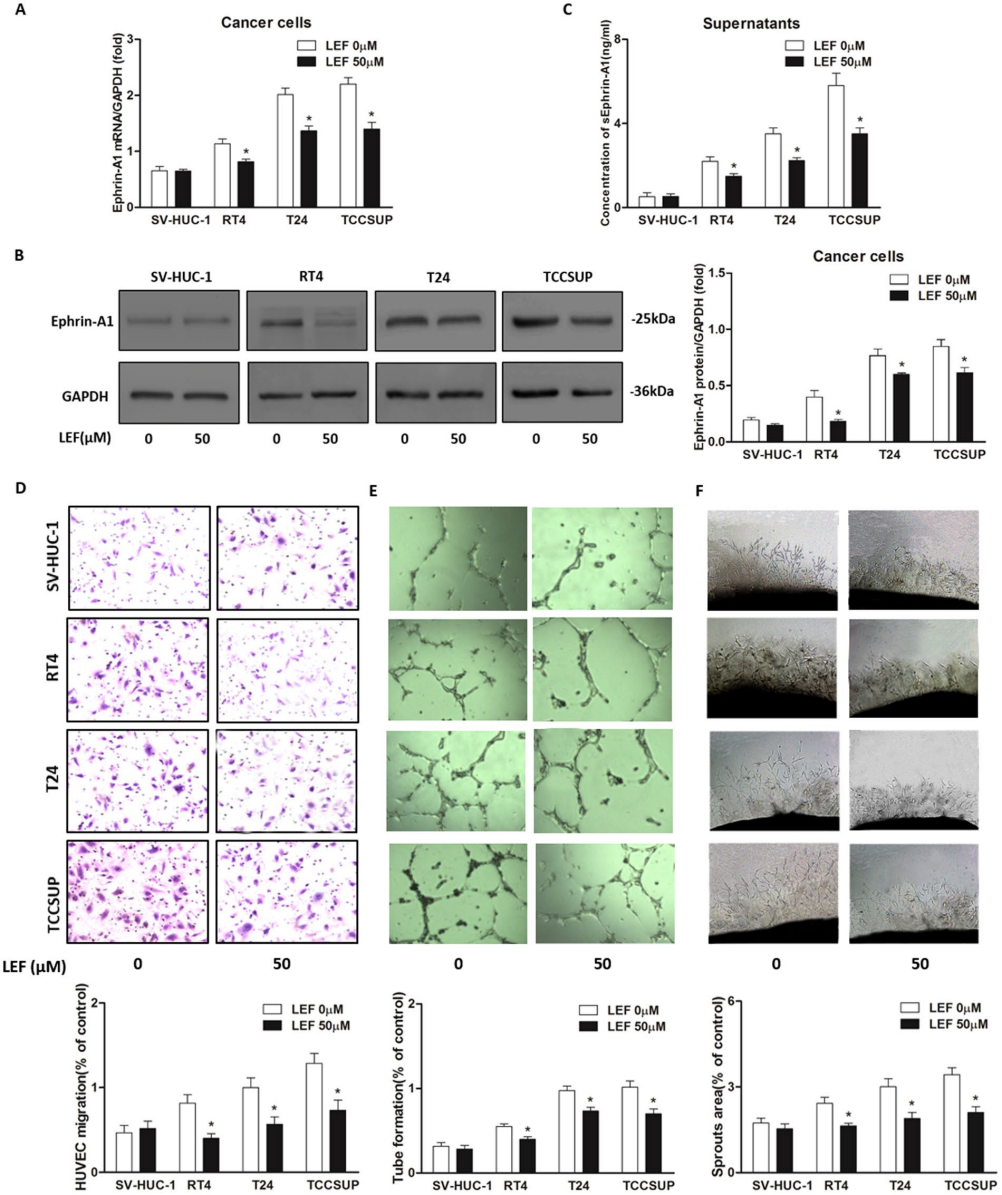
LEF inhibited angiogenesis by modulating ephrin-A1 expression in BCa cells. SV-HUC-1 cells and BCa cells (RT4, T24, TCCSUP cells) were co-cultured with HUVECs respectively under treatment of 50 μM LEF for 48 h. (**A–C**) LEF significantly down-regulated ephrin-A1 mRNA (**A**) expression levels in co-cultured RT4, T24 and TCCSUP cell lines, ephrin-A1 protein (**B**) expression levels in BCa cells and sEphrin-A1 concentrations (**C**) in the supernatants of BCa cells co-cultured with HUVECs (n = 3, respectively; *p < 0.05), while ephrin-A1 expression in co-cultured SVHUC-1 cells and sEphrin-A1 levels in the medium of SVHUC-1 cells and HUVECs co-cultured and treated with LEF did not significantly change. (**D–F**) Transwell assays, tube formation tests and e*x vo* aortic ring angiogenesis assay were performed to determine the effects of LEF on the angiogenesis of HUVECs by using the co-culture supernatants. Migration, tube formation and microvessel sprouting were significantly decreased by supernatants from BCa cell and HUVEC co-cultures treated with LEF (n = 3, respectively) compared to each vehicle control (n = 3, respectively; *p < 0.05). Interestingly, no obvious decrease was observed with the medium from SVHUC-1 cells and HUVECs co-cultured and similarly treated with LEF. Results are expressed as the mean ± S.E.M.

To mimic the micro-environment of tumor tissue, we set up BCa cell and HUVEC co-culture systems using a transwell apparatus. Briefly, RT4, T24, TCCSUP and SV-HUC-1 cells were individually co-cultured with HUVECs. We then investigated the effects of LEF on ephrin-A1 expression in the co-culture systems. The results showed that LEF significantly down-regulated ephrin-A1 mRNA expression levels in co-cultured RT4, T24 and TCCSUP cell lines (*p* < 0.05, Fig. 4A), ephrin-A1 protein expression levels in BCa cells (*p* < 0.05, Fig. 4B) and sEphrin-A1 concentrations in the supernatants of BCa cells co-cultured with HUVECs (*p* < 0.05, Fig. 4C). Interestingly, ephrin-A1 expression in co-cultured SVHUC-1 cells and sEphrin-A1 levels in the medium of SVHUC-1 cells and HUVECs co-cultured and treated with LEF did not significantly change (Fig. 4A–C).

### LEF attenuates angiogenesis *in vitro* and *ex vivo* systems

Since sEphrin-A1 protein levels in co-culture medium could be suppressed by LEF, we performed transwell assays and tube formation tests to determine the effects of LEF on the angiogenesis of HUVECs by using the co-culture supernatants. We observed that the migration and tube formation of HUVECs were significantly decreased by supernatant from BCa cell and HUVEC co-cultures treated with LEF (*p* < 0.05, Fig. 4D,E). Interestingly, no obvious decrease was observed with the medium from SVHUC-1 cells and HUVECs co-cultured and similarly treated with LEF (Fig. 4D,E). We also examined the effects of conditioned media on microvessel sprouting in *ex vivo* aortic ring assays. Similar results to those from the transwell analysis and tube formation assays were obtained (*p* < 0.05, Fig. 4F).

### Overexpression of ephrin-A1 in TCCSUP cells could partially reverse the LEF induced inhibition of ephrin-A1 expression and subsequently the vascularity of HUVECs

To further verify the inhibition of angiogenesis by leflunomide via targeting sEphrin-A1, the effects of LEF on ephrin-A1-overexpressing TCCSUP cell and HUVEC co-cultures on the vascularity of HUVECs was studied. Real-time PCR and western blot analysis revealed that ephrin-A1 expression in TCCSUP cells was significantly down-regulated with LEF treatment (*p* < 0.05, Supplementary Fig. 1A,B). Overexpression of ephrin-A1 in TCCSUP cells could significantly reverse the LEF induced inhibition of ephrin-A1 expression (*p* < 0.05, Supplementary Fig. 1A,B). Similar trends of sEphrin-A1 expression in the supernatants were also obtained (*p* < 0.05, Supplementary Fig. 1C). To confirm the effect of LEF on sEphrin-A1-related angiogenesis, supernatants from empty vector-transfected, ephrin-A1-overexpressing TCCSUP cell and HUVEC co-cultures were added to HUVECs, and vascular functional experiments were performed; similar trends of vascularity as sEphrin-A1 expression levels in supernatants were observed (*p* < 0.05, Supplementary Fig. 1D–F). These findings indicated that the LEF-induced anti-angiogenesis effect was partially mediated by the down-regulation of ephrin-A1 gene expression and subsequent sEphrin-A1 secretion.

### General Observations

BBN is a carcinogen widely used in animal bladder carcinogenesis studies^15^. Mice were administered tap water or tap water containing 0.05% BBN for 20 weeks to induce BCa, and LEF at doses of 10.0 and 20.0 mg/kg/day was administered during and after BBN treatment. As expected, bladder tumors developed in BBN-treated mice, and no bladder tumors were detected in the vehicle control group. During BBN treatment, water intake and body weight were monitored. BBN mice consumed a similar amount of BBN-containing water (Supplementary Fig. 3A), excluding the likelihood that the observed accelerated carcinogenesis in BBN mice was due to excess BBN consumption. The mean body weight of each BBN-induced mouse group with or without LEF treatment was lower than that of normal mice; however, there was no significant difference (Supplementary Fig. 3B). For a better evaluation of LEF toxicity, the mean body weight of normal mice treated with different doses of LEF was also calculated and used as a parameter of toxicity. No significant body weight loss was found among different treatment groups (Supplementary Fig. 3B).

### LEF inhibits the neoplastic progression of BBN-induced bladder carcinogenesis in mice

**Figure 5:**
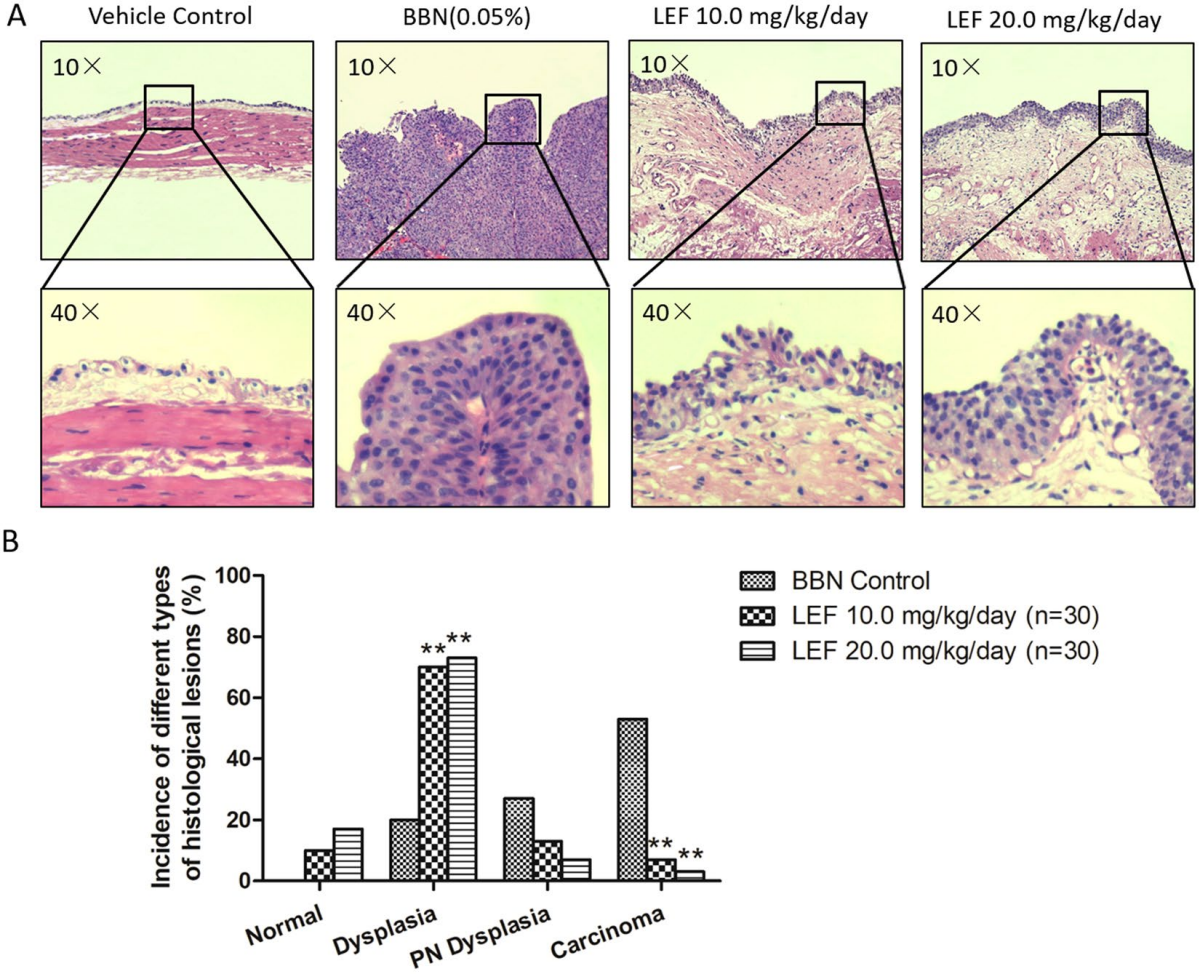
LEF inhibits the neoplastic progression of BBN-induced bladder carcinogenesis in mice. (**A**) Representative photography of HE staining of the bladder tissue from different experimental groups were demonstrated. (**B**) Carcinogenesis was assessed by the pathological incidences. Urothelium was classified as (a) normal urothelial mucosa, characterized by epithelium of <3 layers without any anaplasia; (b) mucosal dysplasia, characterized by epithelium of ≥3 layers with moderate to severe anaplasia with diffused proliferation; (c) papillary/nodular (PN) dysplasia, characterized by moderate or severe anaplastic epithelial lesion of localized cellular proliferation resulting in nodular or papillary forms; (d) urothelial carcinoma, characterized by invasive carcinoma infiltrating the submucosa or muscle layer with transitional-cell carcinoma or undifferentiated features. When mice were fed LEF at doses of 10.0 and 20.0 mg/kg/day (n = 15, respectively), the incidence of urothelial carcinoma significantly decreased compared to that in the BBN control group (n = 15; **p < 0.05). Compared to the BBN control mice, the groups treated with 10.0 and 20.0 mg/kg/day LEF showed a significantly higher incidence of mucosal dysplasia with a concomitant decrease in papillary/nodular dysplasia (**p < 0.05).

To confirm the *in vivo* protective effects of LEF, a detailed histopathological analysis of the neoplastic progression in the BBN–induced bladder carcinogenesis model was performed. As shown in Fig. 5A, the 20-week administration of 0.05% BBN water resulted in the induction of mucosal dysplasia, papillary/nodular dysplasia, and highly aggressive carcinoma of the urinary bladder at the end of the 24-week study. The groups not induced by BBN demonstrated normal histological characteristics. When mice were fed LEF at doses of 10.0 and 20.0 mg/kg/day at the same starting time as BBN administration and continuing until 4 weeks after BBN administration, the incidence of urothelial carcinoma significantly decreased compared to that in the BBN control group (*p* < 0.05, Fig. 5B). Interestingly, compared to the BBN control mice, the groups treated with 10.0 and 20.0 mg/kg/day LEF showed a significantly higher incidence of mucosal dysplasia with a concomitant decrease in papillary/nodular dysplasia (*p* < 0.05, Fig. 5B), and LEF was more effective at a dose of 20.0 mg/kg/day than at 10.0 mg/kg/day; however, no significant difference was observed. Representative photography of HE staining from mice treated with 10.0 or 20.0 mg/kg/day LEF with dysplasia manifestation is shown in Fig. 5A. Taken together, these results clearly showed that both doses of LEF arrested tumor progression at the preneoplastic stage (dysplasia) with a marked reduction in advanced dysplasia and invasive carcinoma in the BBN-induced urinary bladder urothelium.

### LEF suppresses the expression of ephrin-A1 and angiogenesis in BBN-induced bladder carcinogenesis

**Figure 6:**
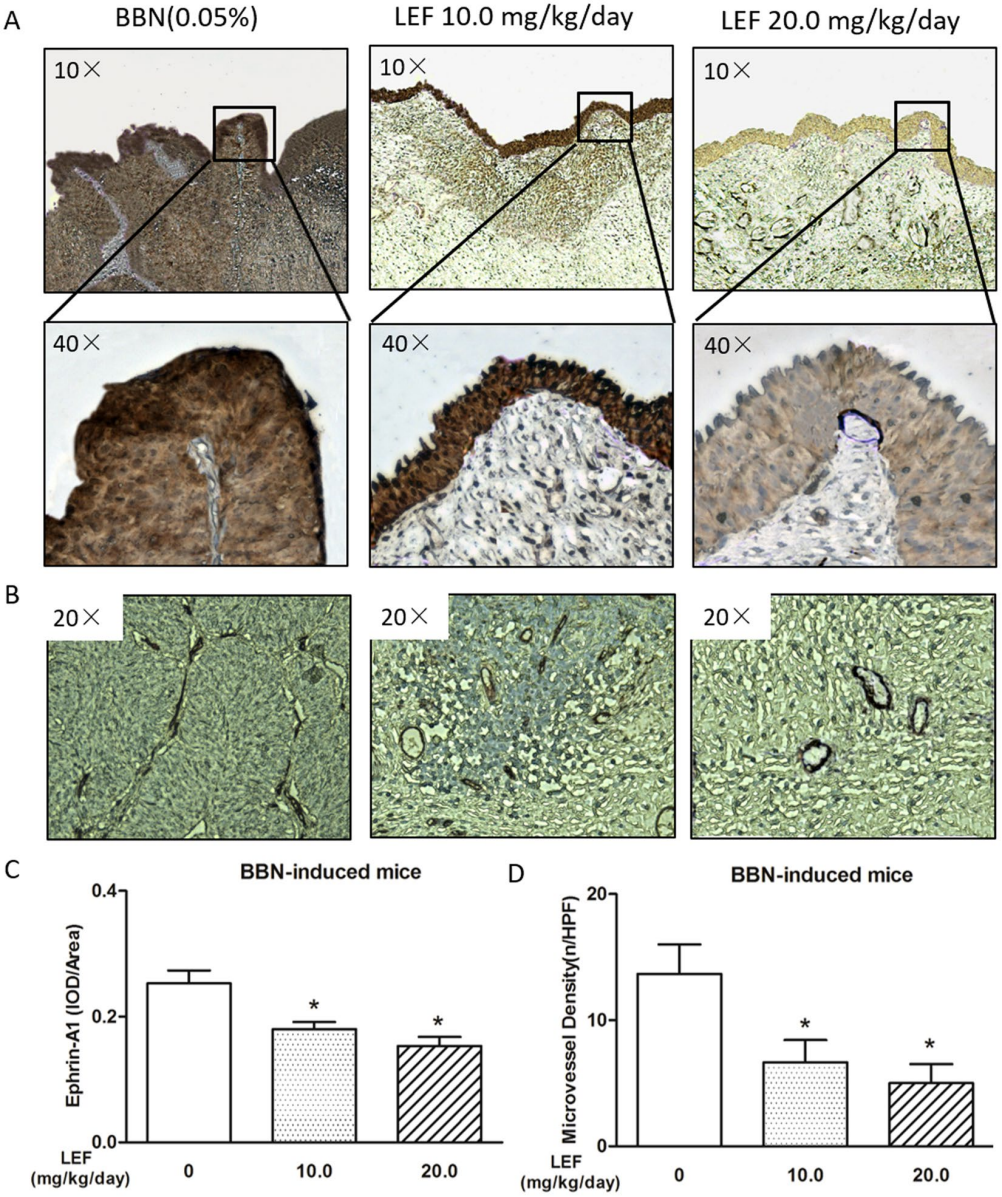
LEF suppresses the expression of ephrin-A1 and microvessel density in BBN-induced bladder carcinogenesis. (**A-B**) Representative photography of immunohistochemical analysis. LEF suppressed ephrin-A1 expression (**A**) and microvessel density (**B**) in BCa tissue. Positive staining appears as brown color. (**C**) The ephrin-A1 protein expression levels were analyzed by calculating the integrated optical density (IOD)/area using Image-Pro Plus. Ephrin-A1 up-regulation was demonstrated in BBN-induced mouse bladder carcinogenesis, and LEF at both doses of 10.0 and 20.0 mg/kg/day could significantly reduce this up-regulation in a dose-dependent manner (n = 15, respectively; *p < 0.05). (**D**) Angiogenesis in tumor tissue was measured via microvessel density (MVD) through CD34 immunostaining. Compared to the BBN control group, MVD significantly decreased upon LEF treatment at doses of 10.0 and 20.0 mg/kg/day in a dose-dependent manner (n = 15, respectively; *p < 0.05). Data are expressed as the mean ± S.E.M.

We performed immunohistochemical analysis to elucidate the molecular mechanism underlying the ability of LEF to suppress bladder carcinogenesis. Consistent with the observation in the BCa cell and HUVEC co-culture model, ephrin-A1 up-regulation was also observed in BBN-induced mouse bladder carcinogenesis *in vivo*, and LEF at both doses of 10.0 and 20.0 mg/kg/day could significantly reduce this up-regulation in a dose-dependent manner (*p* < 0.05, Fig. 6A,C).

Angiogenesis in tumor tissue was measured via microvessel density (MVD) through CD34 immunostaining. LEF-associated anti-angiogenic effects were further confirmed by measuring MVD. Compared to the BBN control group, MVD significantly decreased upon LEF treatment at doses of 10.0 and 20.0 mg/kg/day in a dose-dependent manner (*p* < 0.05, Fig. 6B,D), indicating that LEF may have protective effects through the suppression of ephrin-A1, with a subsequent anti-angiogenic effect in tumor tissues.

### Overexpression of ephrin-A1 could partially reverse the LEF induced inhibition of TCCSUP tumor growth in nude mice

To confirm that the inhibition effect of LEF on angiogenesis was induced via targeting ephrin-A1 *in vivo*, wild-type, empty vector-transfected and ephrin-A1-overexpressing TCCSUP subcutaneous xenografts were generated in nude mice. When the tumors became palpable, the tumor-bearing animals were randomly divided into two groups for LEF treatment at a dosage of 20.0 mg/kg/day and for vehicle treatment. Tumor size was measured every week for 5 weeks. The tumor progression of the wild-type and empty vector-transfected TCCSUP cell groups was similar to each other but in contrast with that in the ephrin-A1-overexpressing TCCSUP group at the end of the experiment (Supplementary Fig. 4A). Tumor progression was significantly suppressed by LEF at a dosage of 20.0 mg/kg/day compared to each vehicle control group, and tumor progression in the ephrin-A1-overexpressing group upon LEF treatment was significantly higher than that in empty vector-transfected TCCSUP group under LEF treatment (Supplementary Fig. 4A). Subcutaneous tumors treated with LEF were markedly smaller than those in each vehicle control group. The average tumor weight at the endpoint in LEF groups was significantly lower compared to that in each vehicle control group (*p* < 0.05, Supplementary Fig. 4B). The average tumor weight in the ephrin-A1-overexpressing group treated with LEF were significantly higher than that in empty vector-transfected TCCSUP group upon LEF treatment (*p* < 0.05, Supplementary Fig. 4B).

### Overexpression of ephrin-A1 could partially reverse the LEF induced inhibition of ephrin-A1 expression and subsequently angiogenesis in TCCSUP tumor xenografts

Ephrin-A1 expression and MVD in tumor xenograft tissues were evaluated by immunohistochemical analysis. The staining intensity for ephrin-A1 was strikingly reduced in LEF treatment groups at a dosage of 20.0 mg/kg/day compared to that in each vehicle control group (*p* < 0.05, Supplementary Fig. 5). The immunostaining for ephrin-A1 in the ephrin-A1-overexpressing group treated with LEF was significantly higher than that in empty vector-transfected TCCSUP group under LEF treatment (*p* < 0.05, Supplementary Fig. 5). Indeed, a changing trend of MVD similar to that of ephrin-A1 expression in tumor tissue was also observed (*p* < 0.05, Supplementary Fig. 6).

## Discussion

Angiogenesis is one of the most important factors in BCa progression^16^. BCa cells in tumor tissue may affect the vascular endothelium and therefore the interaction between BCa cells and microvascular endothelial cells via angiogenesis-related cytokines that play a crucial role in disease progression. However, the molecules and mechanisms contributing to angiogenesis in BCa remain unknown. Membrane-anchored ephrin-A1, which is overexpressed in BCa^5^, has previously been reported to have a critical function in inducing tumor angiogenesis through its receptor EphA2 expressed on endothelial cells^7^, and the important physiological functions of ephrin-A1 are largely dependent on cell–cell contact^4,17^. In the present study, we found that the soluble form of ephrin-A1 was significantly up-regulated in the supernatants of BCa cells co-cultured with HUVECs compared with that in SV-HUC-1 co-cultured with HUVECs, and the corresponding EphA2 expression on endothelial cells was down-regulated, confirming that this soluble ephrin-A1 is functional as the downstream mediator of EphA2 on endotheliocytes. The existence of this soluble form of ephrin-A1 was consistent with the results of a previous study in other tissues^11^. Importantly, this enhanced functional protein may be biologically active as an angiogenic factor causing increased vascularity, as demonstrated by transwell migration, tube formation and microvessel sprouting assays, due to internalization and thus activation of EphA2 on HUVECs. Surprisingly, higher ephrin-A1 expression (including both forms of ephrin-A1) in BCa cell lines was correlated with lower differentiation levels, leading to the assumption that ephrin-A1 expression level may be associated with the degree of malignancy. Therefore, ephrin-A1 might be used as a biomarker for BCa prognosis, which has been suggested in other types of cancer^18,19^.

To precisely evaluate the regulatory role of sEphrin-A1 in BCa angiogenesis, we examined its expression levels and functional status by using interventions such as ephrin-A1 stimulation, overexpression and knockdown *in vitro*, as well as ephrin-A1 overexpression *in vivo*. Owing to the most significant up-regulation of ephrin-A1 expression, the TCCSUP cell line was applied as the experimental object. The results showed that sEphrin-A1 expression in the supernatant may be significantly up- or down-regulated through ephrin-A1 stimulation/overexpression or knockdown in TCCSUP cells, and vascularity as determined via transwell migration, tube formation *in vitro* and aortic ring assays *ex vivo* showed similar changes upon treatment with supernatants from cells in which ephrin-A1 levels or activity were altered. The opposite regulation of EphA2 protein expression on HUVECs suggests the activation of this receptor by sEphrin-A1 secreted from BCa cells. These results, suggesting that ephrin-A1 plays an important role in inducing angiogenesis, were consistent with those of Brantley-Sieders *et al*.^7^ but were in contrast with the observations of Ojima *et al*.^20^, potentially owing to the fact that the soluble form of ephrin-A1 was mainly examined in the present study, and the tissue contexts were different.

Due to the important role of the sEphrin-Eph system in inducing angiogenesis in BCa, precise-treatments could lead to BCa disease amelioration. However, monoclonal antibodies, recombinant proteins, or small molecules (derivatives of lithocholic acid) targeting the ephrin-Eph system suffer from significant proteolytic degradation^21,22^ or poor physicochemical properties^23^; therefore, new drugs targeting the ephrin-Eph system are needed. However, although surgical therapy is effective in the treatment of non-invasive BCa, a majority of BCa cases initially present with a low-grade tumor confined to the mucosa with a high recurrence rate. Up to one-third of these non-muscle invasive cancers demonstrate disease progression to more invasive or higher-grade tumors. Developing novel therapeutic agents that inhibit the molecular pathways responsible for angiogenesis is crucial for BCa therapy. LEF has attracted worldwide attention as one of the most promising effective medications against autoimmune diseases. LEF is a novel promising therapeutic agent for cancer^24,25^ with anti-angiogenic potential^14^. The present investigation showed that this anti-angiogenic effect of LEF was less dependent on cytotoxicity, as the effective doses were far below the dosage that led to a reduction in cell viability. Interestingly, this current study demonstrated that the great clinical potential of LEF as an anti-tumor agent is that it can inhibit angiogenesis by specifically targeting the sEphrin/Eph system in BCa cell and HUVEC co-cultures in a dose-dependent and paracrine manner. This effect was also confirmed in the BBN-induced bladder carcinogenesis model *in vivo*. LEF administration can inhibit the neoplastic progression of BBN-induced mouse bladder carcinogenesis by targeting angiogenesis via the rescue of the deregulated expression of ephrin-A1. To further verify that the inhibition of angiogenesis by leflunomide was specifically mediated by targeting sEphrin-A1, ephrin-A1 overexpression experiments were performed both *in vivo* and *in vitro*, and ephrin-A1 overexpression could partially reverse the LEF-induced suppression of angiogenesis and subsequent tumor growth inhibition.

In addition, although ephrin-A1 expression by BCa cells was suppressed by LEF, ephrin-A1 expression in normal bladder cells (i.e., SVHUC-1) was not significantly influenced. This favorable anti-angiogenesis profile of LEF enables the formulation of drug applications with lower toxicity and promotes improvements in drug safety during clinical use and during combination therapy; these data provide new insight into the anti-angiogenesis activities of LEF, although the mechanisms remain to be investigated.

In summary, LEF can minimize angiogenesis through inhibiting the sEphrin-A1/EphA2 system (Supplementary Fig. 7). We now continue to research LEF binding proteins and novel targets in BCa to fully understand the effects of this treatment, and the precise mechanism of LEF treatment on BCa angiogenesis warrants further study. We should also determine how this functional system cross-talks with other processes, such as NF-κB-related apoptosis^26^, which are involved in tumor failure.

In closing, we observed that the sEphrin-A1/EphA2 system is systematically up-regulated in BCa tissues both *in vitro* and *in vivo*. Moreover, LEF may have an anti-angiogenesis effect on BCa cells and tumor tissue in pre-clinical mouse models through this functional system and may serve as a valuable agent for the development of novel BCa precision therapeutic strategies, either as a monotherapy or a combination therapy.

## Materials and Methods

### Cells

BCa cell lines (RT4, T24, and TCCSUP) and immortalized normal urothelial cells (SV-HUC-1) were obtained from American Type Culture Collection (Manassas, VA, USA). BCa cells were cultured in RPMI 1640 containing 10% FBS. SV-HUC-1 cells were cultured in F12K medium with 10% FBS. HUVECs were purchased from ScienCell Research Laboratories (6076 Corte Del Cedro, Carlsbad, CA, USA) and were maintained in Endothelial Cell Growth Medium (EGM)-2 from the same company.

### Co-culture of BCa cells and HUVECs

BCa cells (RT4, T24, and TCCSUP) and SV-HUC-1 cells were individually co-cultured with HUVECs using a six-well transwell apparatus. Briefly, RT4, T24, TCCSUP and SV-HUC-1 (5 × 10^5^ cells/ml) cells were seeded in RPMI 1640 containing 10% FBS and allowed to adhere overnight. Cells were then washed with serum-free RPMI 1640, and suspensions of HUVECs (ranging from 3 × 10^5^ cells/ml to 4 × 10^5^ cells/ml) were added into the upper chamber of the transwell apparatus (Costar, New York, USA). LEF (Sigma, L5025) at a final concentration of 50 μM was administered to bladder cells and HUVEC co-cultures. After culture in RPMI 1640 with 1% FBS for 48 hours, the supernatants of the co-cultures were harvested. Part of each supernatant was used for subsequent transwell assays, tube formation tests, *ex vivo* aortic ring angiogenesis assays, and western blot analysis for soluble ephrin-A1 expression. The remaining supernatant was frozen at −20 °C until further analysis by a commercial ELISA kit for ephrin-A1 (E-EL-H0875c, Elabscience, China).

### Transwell assays

Migration activity was measured using a 24-well transwell apparatus (Costar, New York, USA; pore size, 8.0-μm), and 600 µL of supernatant from the co-culture was placed in the lower chamber. HUVECs (2 × 10^4^ cells in 200 µL of serum-free RPMI 1640) were added to the upper chamber and incubated for 6 hours. Non-migrating cells on the upper side of the filters were removed. Cells that migrated to the lower phase were stained. Images were obtained via microscopy (LEICA DMi8), and the number of cells that migrated to the lower side was quantified at 100 × magnification.

### HUVEC tube formation *in vitro*

After thawing on ice, 50 μl of Matrigel (Corning, New York, USA) was plated in each well of a 96-well culture plate and allowed to gel for 30 minutes at 37 °C; then, 50 μl of fresh supernatant from the co-culture was added to each well. HUVECs were then re-suspended at a concentration of 2 × 10^5^ cells/ml in RPMI 1640. Next, 100 μl of cell suspension was added to each chamber. After 6 hours of incubation, endothelial cell tube formation was assessed using phase-contrast microscopy and then photographed. The morphology of the tube-like structures in the well was assessed and quantified at 50× magnification.

### Mouse aortic ring angiogenesis assay

The *ex vivo* aortic ring assay was performed as previously described^27^. Briefly, 24-well culture plates were coated with 200 μl of growth factor-reduced (GFR) Matrigel (Corning, New York, USA). Thoracic aortas were removed from 6- to 8-week-old C57BL/6 mice (SPF, Laboratory Animal Center of the Second Affiliated Hospital of Harbin Medical University) and sectioned into 1-mm-long aortic rings. Each ring was placed in the pre-coated well and covered with an additional 200 μl of Matrigel. Aortic rings were incubated for 3 days in EGM-2. After verifying sprouts from the aortic rings, the EGM-2 was replaced with 500 μl of fresh supernatant from bladder cell and HUVEC co-cultures with or without LEF treatment, and the rings were further incubated for 3 days. Microvessel outgrowths were examined with microscopy (LEICA DMi8) and photographed (50× magnification). The area of outgrowth was analyzed using ImageJ software.

### Stimulation

The expression of ephrin-A1 increased most significantly in the TCCSUP cell line; therefore, TCCSUP cell and HUVEC co-cultures were used to further evaluate the biologic activity of soluble ephrin-A1 in the supernatants. The TCCSUP cell and HUVEC co-culture system was established as described above and treated with fresh culture medium containing recombinant human ephrin-A1-Fc protein (R&D Systems, Minneapolis, USA) at a concentration of 5 μg/ml^28^. Supernatants and co-cultured HUVECs were harvested at 48 hour after ephrin-A1 stimulation.

### Overexpression

Ephrin-A1 overexpression was performed using complete cDNA as previously described^29^. Recombinant lentiviruses were produced in human embryonic kidney cells (HEK-293T). T24 cells were cultured in 6-well plates at a density of 9.5 × 10^5^ cells/well and transfected with the ephrin-A1 vectors at a multiplicity of infection (m.o.i.) of 100. After three days of transfection, the efficiency of overexpression was determined by RT-PCR and western blot analysis. The transfected cells and HUVEC co-culture system was then established as described above and treated with LEF at a concentration of 50 μM. Supernatants were harvested 48 hours later for further experiments.

### RNA interference analysis

Ephrin-A1 knockdown experiments were performed by transfecting specific ephrin-A1 small interfering RNA (siRNA) into TCCSUP cells in the lower chamber. The siRNA sequences targeting ephrin-A1 were forward 5′-CCATACATGTGCAGCTGAA-3′ and reverse 5′-CCAGTACACGTTCGATGAA-3′. The following procedures were performed: TCCSUP cells were plated in the lower chamber at a density of 5 × 10^5^ cells/well, and the cells were transfected with siRNA using Lipofectamine 2000 (Invitrogen, Carlsbad, CA, USA). The final concentration of the siRNA duplex was 100 nM. At 24 hours after transfection, 75% knockdown efficiency was achieved, as determined by real-time PCR and western blot analysis. The medium was replaced with RPMI 1640 with 1% FBS, and HUVECs were simultaneously added to the upper chamber. Supernatants and HUVECs co-cultured were harvested 48 hours later for further experiments.

### Cell viability assay

Cell lines (SV-HUC-1, RT4, T24 and TCCSUP) were seeded onto 96-well plates at a density of 10^4^/well and subsequently treated with LEF at various concentrations (0, 12.5, 25, 50, 100 and 200 μM). At 0, 24, 48 and 96 hours, 10 μl of CCK8 (Beyotime Institute of Biotechnology, Shanghai China) was added into each well, and the plates were incubated at 37 °C for 2 hours. The optical density (OD) was detected, the proliferation curve was drawn, and cell viability was determined.

### Real-time quantitative PCR analysis

Total RNA was extracted from bladder cells, converted to cDNA and subjected to real-time PCR amplification using GoTaq qPCR Master Mix (Promega, Beijing, China). Specific amplification was performed using *ephrin-A1* gene primers (forward primer: 5′-CGGAATGAGGACTACACCATACATGTGCAGC-3′; reverse primer: 5′-AAGCAGCGGTCTTCATGCTGGTGGATGGGTT-3′). *GAPDH* (forward primer: 5′-ATGGTGGGTATGGGTCAGAAG-3′; reverse primer: 5′-TGGCTGGGGTGTTGAAGGTC-3′) was used as an internal control. The results were evaluated using the 2^−ΔΔCt^ method as previously described^30^.

### Western blot analysis

The ephrin-A1 protein levels in bladder cells and co-culture supernatants were determined by western blot analysis using rabbit polyclonal anti-ephrin-A1 antibody (catalog no. ab199697, Abcam, Cambridge, MA). EphA2 protein in HUVECs was detected by western blot analysis using mouse monoclonal anti-EphA2 antibody (Sigma, St Louis, MO, USA). The procedures were performed as previously described^30^.

### BBN-induced BCa mouse model and LEF therapy

All animal experiments were performed according to the National Institutes of Health Guide for the Care and Use of Laboratory Animals, and the protocols were approved by the Institutional Animal Care and Use Committee of Harbin Medical University. N-Butyl-N-(4-hydroxybutyl)-nitrosamine (BBN, TCI America, B0938) was administered to mice via drinking water following the protocol described before^31^. Briefly, BBN was dissolved in tap water at a concentration of 0.05%. Male C57BL/6 mice (6 weeks old, SPF, Laboratory Animal Center of the Second Affiliated Hospital of Harbin Medical University) were administered drinking water containing 0.05% BBN twice a week for 20 weeks, and LEF treatments were administered from the first BBN administration and lasted for 24 weeks. The mice were randomly divided into four groups (n = 15): vehicle control group (given tap water), BBN control group (given BBN-containing water) and LEF groups (given BBN-containing water and gavaged with 10.0 and 20.0 mg/kg/day LEF, 6 days/week for 24 weeks starting from the first BBN administration). For the evaluation of LEF toxicity, normal mice were gavaged with LEF at doses of 10.0 and 20.0 mg/kg/day. The experimental design is also shown in Supplementary Fig. 2. The body weight and water intake were recorded every three days.

### Tumor xenografts

Wild-type, empty vector-transfected and ephrin-A1-overexpressing TCCSUP cells were mixed with an equal volume of Matrigel, and the total volume of each injection was 0.1 ml. The cell number was 2.5 × 10^6^. The mixture of Matrigel and cells of each type was injected subcutaneously into the flank of male nude mice (4–6 weeks old, SPF, Laboratory Animal Center of the Second Affiliated Hospital of Harbin Medical University). At 10 days after cell inoculation, the animals under different cell treatments with palpable tumors were further randomly divided into two groups (n = 6): vehicle control group (given PBS) and LEF treatment group (gavaged with 20.0 mg/kg/day LEF for 5 weeks). Tumor volume was calculated by the formula tumor volume [mm^3^] = (length [mm]) × (width [mm])^2^/2. At the endpoint, tumors were excised and weighed. Paraffin-embedded tissues were used for histological and immunohistochemical analysis.

### Histological analysis

Tumor tissues obtained from the sacrificed mice were stained with hematoxylin and eosin (H&E staining) for histopathological evaluation. A detailed histopathological analysis of the neoplastic progression in the BBN-induced bladder carcinogenesis was performed as previously described^32^. Briefly, H&E-stained sections were examined and classified as (a) normal urothelial mucosa, characterized by epithelium of <3 layers without any anaplasia; (b) mucosal dysplasia, characterized by epithelium of ≥3 layers with moderate to severe anaplasia with diffused proliferation; (c) papillary/nodular dysplasia, characterized by moderate or severe anaplastic epithelial lesion of localized cellular proliferation resulting in nodular or papillary forms; and (d) urothelial carcinoma, characterized by invasive carcinoma infiltrating the submucosa or muscle layer with transitional cell carcinoma or undifferentiated features.

### Immunohistochemical analysis

The immunohistochemical analysis was performed as previously described, with some modifications^30^. Paraffin-embedded tissues were incubated with anti-ephrin-A1 antibody (catalog no. ab199697, Abcam, Cambridge, MA). Subsequently, the sections were stained using a polymer HRP detection system (PV9001, ZSGB-BIO, Beijing, China) and were visualized with a DAB peroxidase substrate kit (ZLI-9017, ZSGB-BIO, Beijing, China). The immunostained images were captured using a microscope (LEICA DMi8). Protein expression levels were analyzed by calculating the integrated optical density (IOD) per stained area using Image-Pro Plus version 6.0.

Anti-CD34 antibody (BA3414, Boster, Wuhan, China) was used to identify endothelium^31,33^, and the MVD quantification was performed in accordance with the counting method of Weidner *et al*.^34^. Briefly, the five most vascularized areas with the greatest number of tumor microvessels were chosen at low power fields (100× magnification). The microvessels were then counted on a 200× high power field within the designated neovascular region. The MVD was defined as the number of vessels per high power field (200× magnification).

### Data Availability

All data generated or analysed during this study are included in this article and its supplementary information files.

### Statistical analysis

The data were expressed as means ± S.E.M. Statistical analysis was conducted with paired t test or ANOVA as appropriate by using SPSS version 17.0 software. Values of *p* < 0.05 were considered statistically significant.

### Ethical approval information

Harbin Medical University Ethics Committee Board.

## Electronic supplementary material

Supplementary Information
