# Supplementary material for: Inhibition of angiogenesis by leflunomide via targeting the soluble ephrin-A1/EphA2 system in bladder cancer
Source: Sci Rep. 2018 Jan 24;8:1539. doi: 10.1038/s41598-018-19788-y (PMC5784165; doi:10.1038/s41598-018-19788-y)
Supplement: Supplementary file 1 — Supplementary Information [file 41598_2018_19788_MOESM1_ESM.doc]

**Inhibition of angiogenesis by leflunomide via targeting soluble ephrin-A1/EphA2 system in bladder cancer**

Maolin Chu ^1*^, Chunying Zhang^1*^

^1^Department of Urology, The Second Affiliated Hospital, Harbin Medical University, 246 Xuefu St., Nan Gang District, Harbin, China.

*Correspondence should be addressed to: Chunying Zhang (Telephone:86 451 86296753, Fax: 86 451 86296753, Email: cyzhanghlj@QQ.com) or Maolin Chu (Email: chumaolin@163.com ).

**
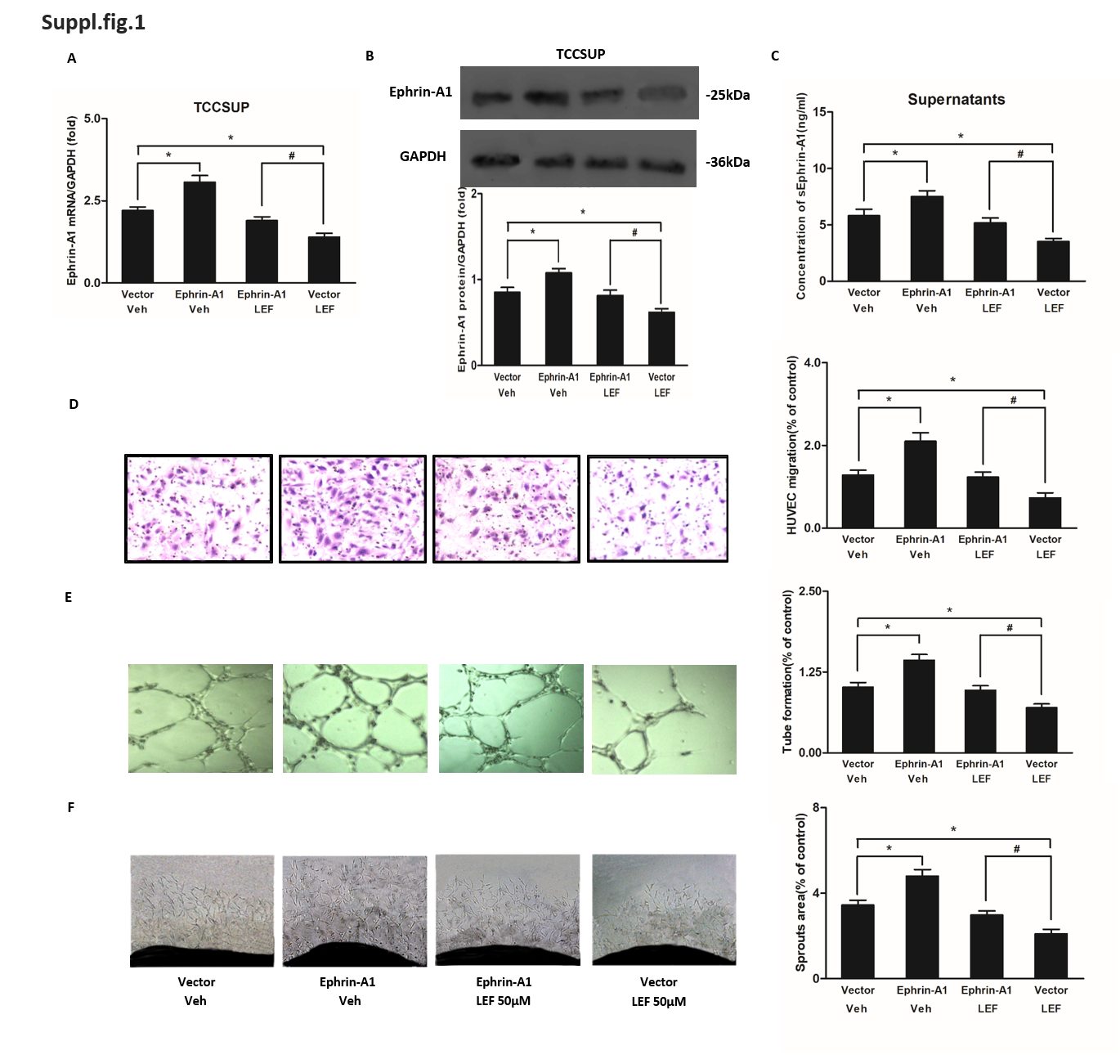
**

**Supplementary Figure 1. Overexpression of ephrin-A1 in TCCSUP cells could partially reverse the LEF induced inhibition of ephrin-A1 expression and subsequently the vascularity of HUVECs. (A-C)** Real-time PCR (A) and western blot analysis (B) revealed that significant increase in the expression of ephrin-A1 were obtained in ephrin-A1-overexpressing TCCSUP cells compared to empty vector-transfected cells (n=3, respectively; **p*<0.05 versus empty vector control). Ephrin-A1 expression in TCCSUP cells was significantly down-regulated with LEF treatment (n=3, respectively; **p*<0.05 versus empty vector control). Overexpression of ephrin-A1 in TCCSUP cells could significantly reverse the LEF induced inhibition of ephrin-A1 expression (n=3, respectively; #*p*<0.05). ELISA (C) revealed similar trends of sEphrin-A1 expression in the supernatants. **(D-F)** Supernatants from empty vector-transfected, ephrin-A1-overexpressing TCCSUP cell and HUVEC co-cultures were added to HUVECs, transwell assay (D; n=3), tube formation test (E; n=3) and *ex vo* aortic ring angiogenesis assay (F; n=3) were performed; similar trends of vascularity as sEphrin-A1 expression levels in supernatants were observed. Results are expressed as the mean ± S.E.M. Veh = vehicle control. Vector = empty vector control. Ephrin-A1= ephrin-A1-overexpressing TCCSUP group.


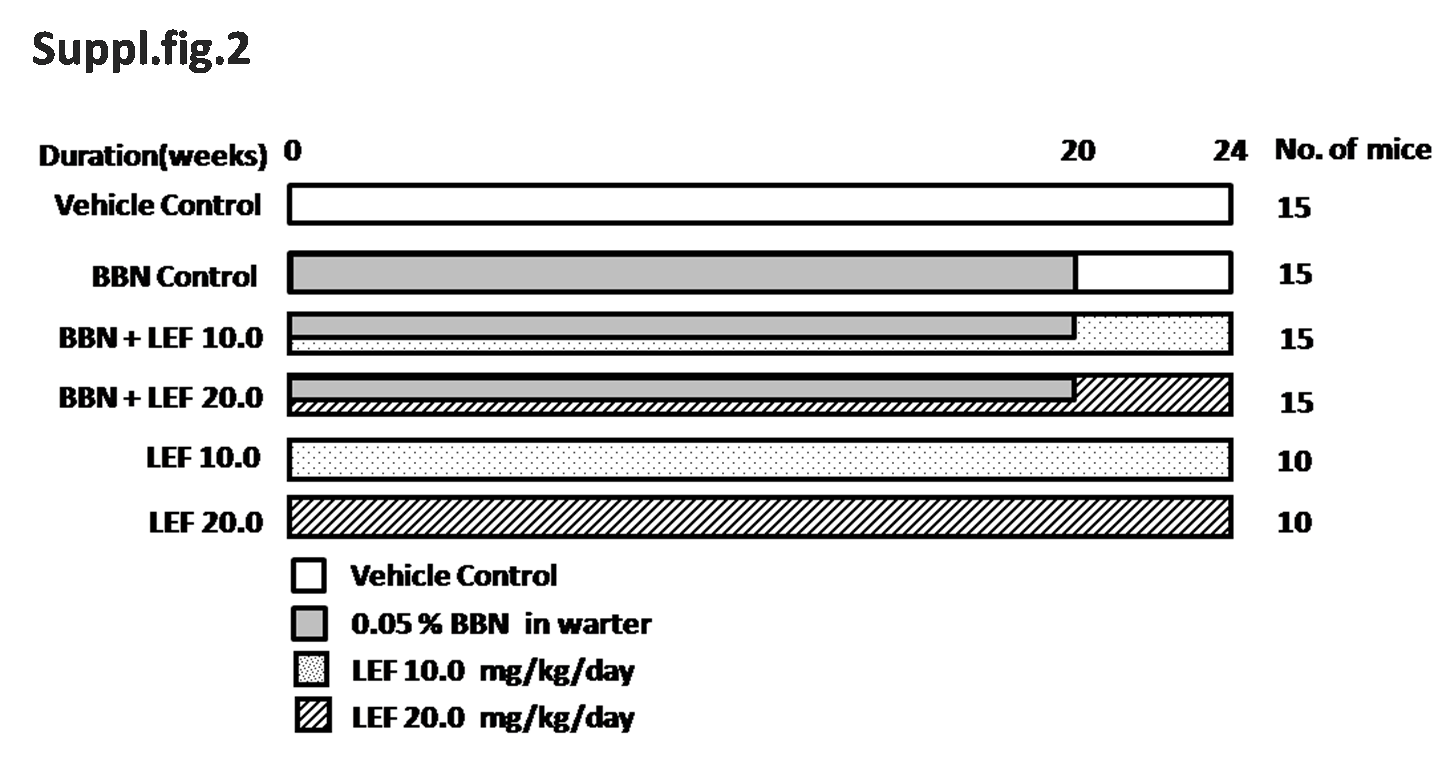


**Supplementary Figure 2.** **Experimental design to study the effect of LEF on BBN–induced bladder carcinogenesis in mice.** Male C57BL/6 mice were randomly divided into six groups. Bladder cancer was induced by administration of drinking tap water containing 0.05% BBN twice a week for 20 weeks (15 mice in each group). BBN-induced mice in LEF treatment groups were also gavaged with LEF 10.0 mg/kg/day and 20.0 mg/kg/day respectively, 6 days/week for 24 weeks, starting from the first BBN administration until 4 weeks after BBN termination. Mice in normal control group (n=15) and BBN control group (n=15) were gavaged with tap water for the same time period. On the other hand, for evaluation of LEF toxicity, normal mice gavaged with LEF at doses of 10.0 and 20.0 mg/kg/day (n=10, respectively) were also gavaged for this 24-week period.

**
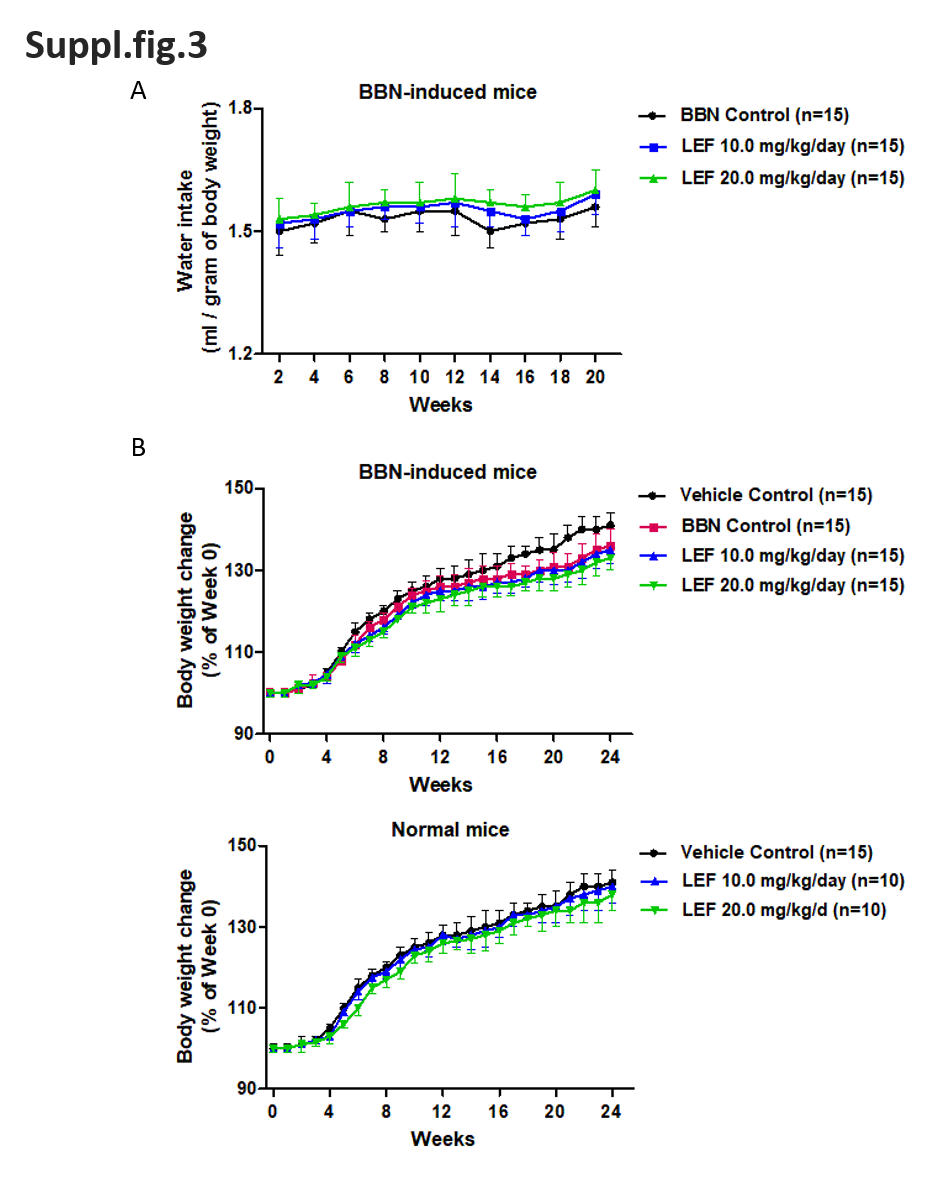
**

**Supplementary Figure 3.** **Water intake and body weight.** During BBN treatment, water intake and body weight were monitored. **(A)** BBN mice consumed a similar amount of BBN-containing water, excluding the likelihood that observed accelerated carcinogenesis in BBN mice was due to excess BBN consumption. (B) The mean body weight of each BBN-induced group with or without LEF treatment was lower than that of normal group; however, there was no significant difference. For a better evaluation of LEF toxicity, mean body weight of normal mice treated with different doses of LEF was also calculated and used as a parameter of toxicity. No significant body weight loss was found among different treatment groups. Results are expressed as the mean ± S.E.M.


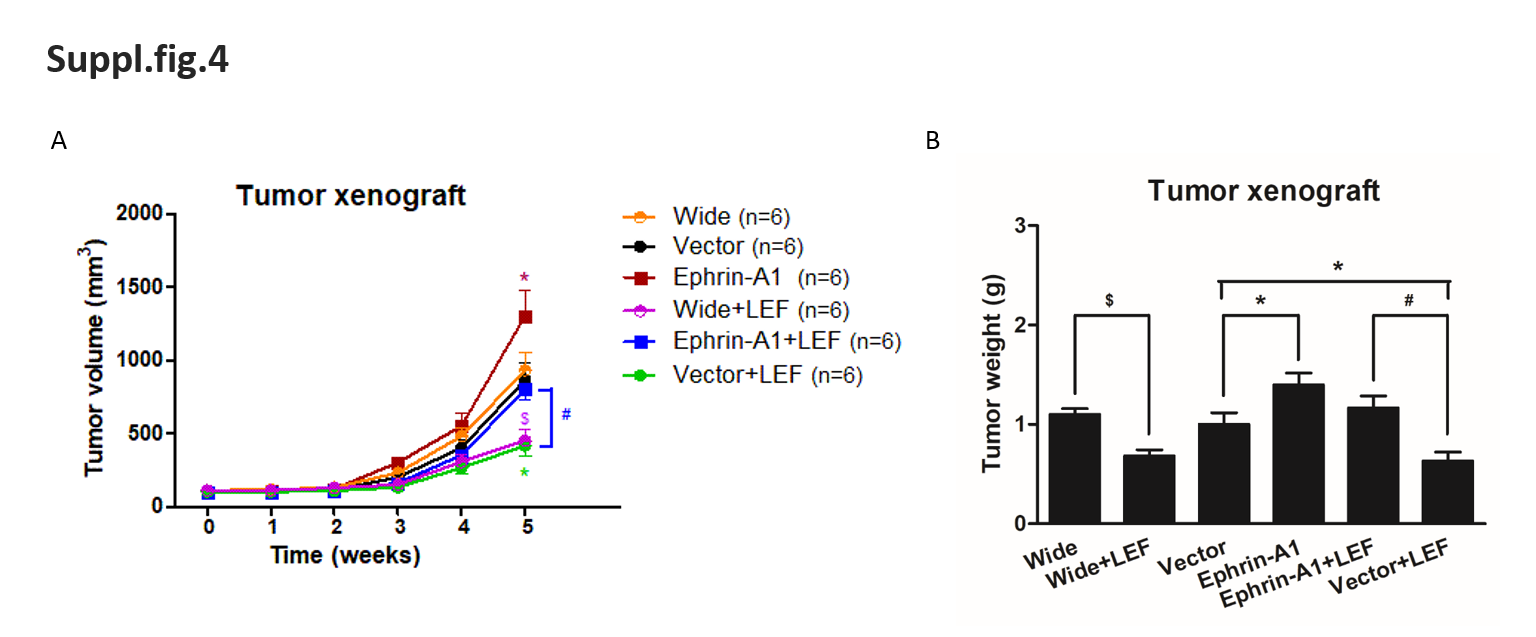


**Supplementary Figure 4. Overexpression of ephrin-A1 could partially reverse the LEF induced inhibition of TCCSUP tumor growth in nude mice.** **(A)** Tumor progression was significantly suppressed by LEF at a dosage of 20.0 mg/kg/day compared to each vehicle control group (n=6, respectively; $*p*<0.05 versus wide control group, **p*<0.05 versus empty vector-transfected TCCSUP group), and tumor progression in the ephrin-A1-overexpressing group upon LEF treatment was significantly higher than that in empty vector-transfected TCCSUP group under LEF treatment (n=6, respectively; #*p*<0.05). **(B)** The average tumor weight at the endpoint in LEF groups was significantly lower compared to that in each vehicle control group (n=6, respectively; $*p*<0.05 versus wide control group, **p*<0.05 versus empty vector-transfected TCCSUP group). The average tumor weight in the ephrin-A1-overexpressing group treated with LEF were significantly higher than those in empty vector-transfected TCCSUP group upon LEF treatment (n=6, respectively; #*p*<0.05). Results are expressed as the mean ± S.E.M. Wide = wide control group. Vector = empty vector-transfected TCCSUP group. Ephrin-A1= ephrin-A1-overexpressing TCCSUP group.

**
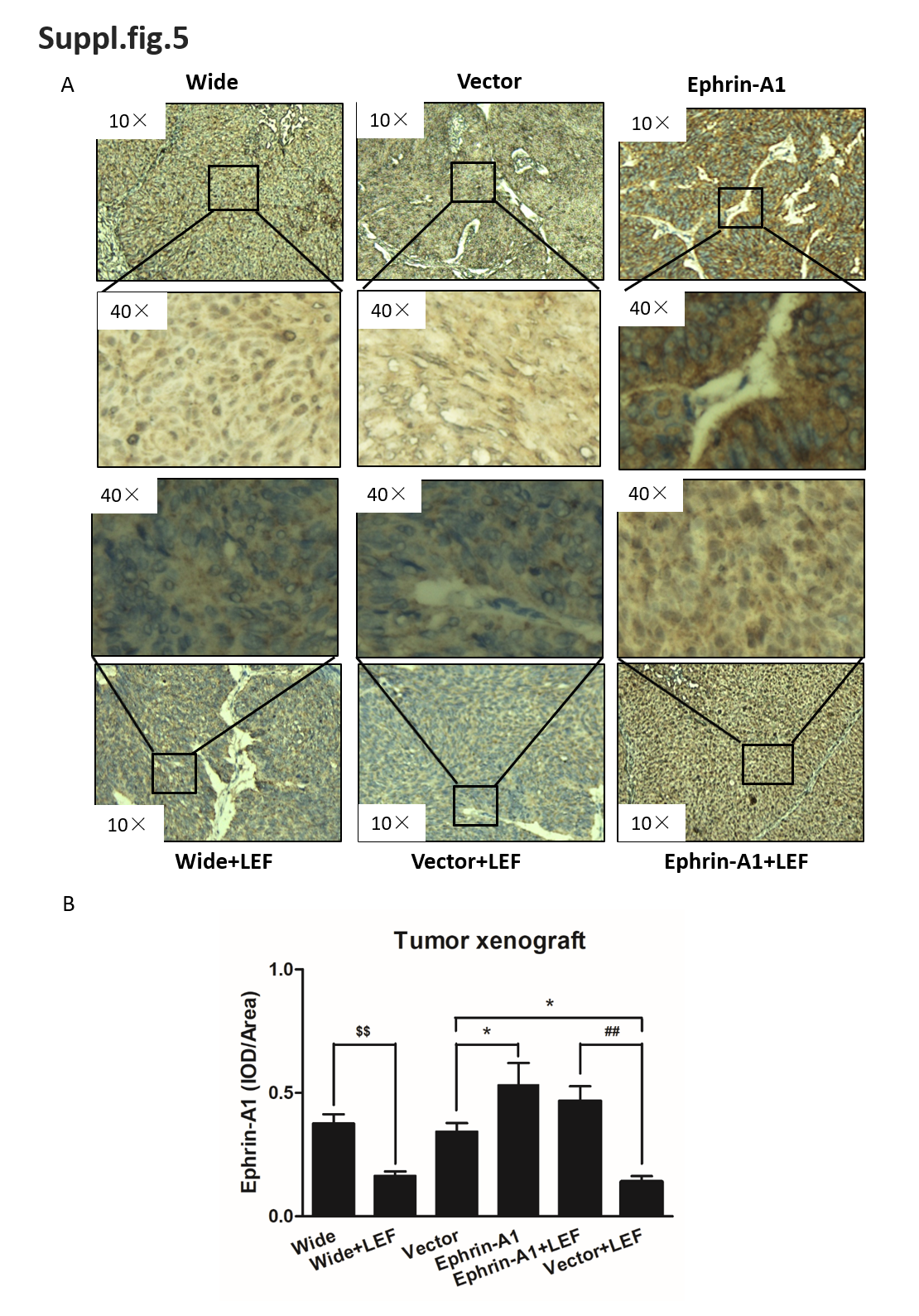
**

**Supplementary Figure 5. Overexpression of ephrin-A1 could partially reverse the LEF induced inhibition of ephrin-A1 expression in TCCSUP tumor** **xenografts.** The ephrin-A1 protein expression levels were analyzed by calculating the integrated optical density (IOD)/area. The staining intensity **(A)** as well as IOD/area value **(B)** for ephrin-A1 were strikingly reduced in LEF treatment groups at a dosage of 20.0 mg/kg/day compared to that in each vehicle control group (n=6, respectively; $$*p*<0.01 versus wide control group, **p*<0.05 versus empty vector-transfected TCCSUP group). The immunostaining for ephrin-A1 in the ephrin-A1-overexpressing group treated with LEF was significantly higher than that in empty vector-transfected TCCSUP group under LEF treatment (n=6, respectively; ##*p*<0.01). Results are expressed as the mean ± S.E.M. Wide = wide control group. Vector = empty vector-transfected TCCSUP group. Ephrin-A1= ephrin-A1-overexpressing TCCSUP group.

**
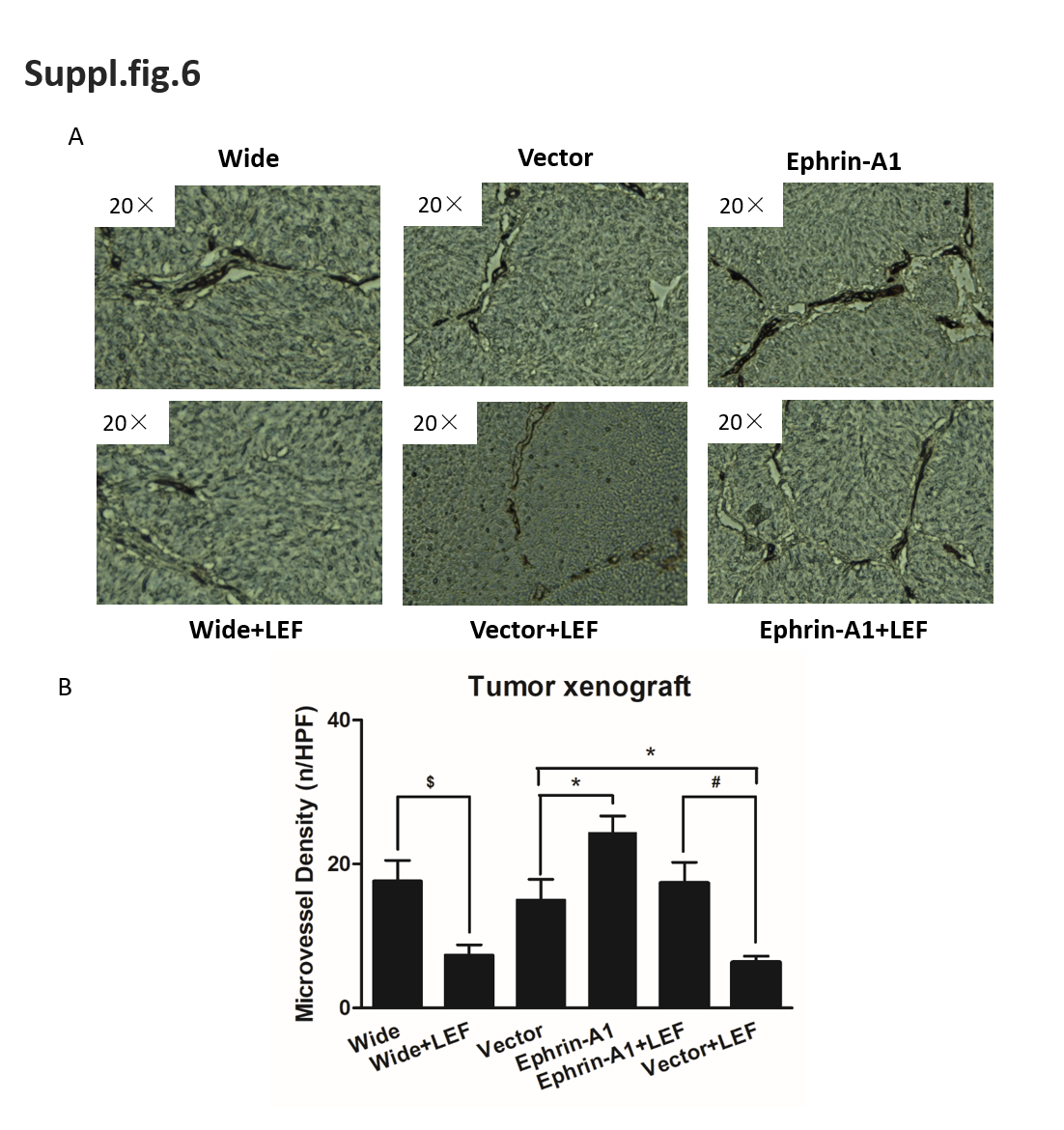
**

**Supplementary Figure 6. Overexpression of ephrin-A1 could partially reverse the LEF induced inhibition of angiogenesis in TCCSUP tumor xenografts. (A)** Representative photography of angiogenesis in tumor tissue were shown. **(B)** MVD was strikingly reduced in LEF treatment groups at a dosage of 20.0 mg/kg/day compared to that in each vehicle control group (n=6, respectively; $*p*<0.05 versus wide control group, **p*<0.05 versus empty vector-transfected TCCSUP group). MVD in the ephrin-A1-overexpressing group treated with LEF was significantly higher than that in empty vector-transfected TCCSUP group under LEF treatment (n=6, respectively; #*p*<0.05). Results are expressed as the mean ± S.E.M. Wide = wide control group. Vector = empty vector-transfected TCCSUP group. Ephrin-A1= ephrin-A1-overexpressing TCCSUP group.


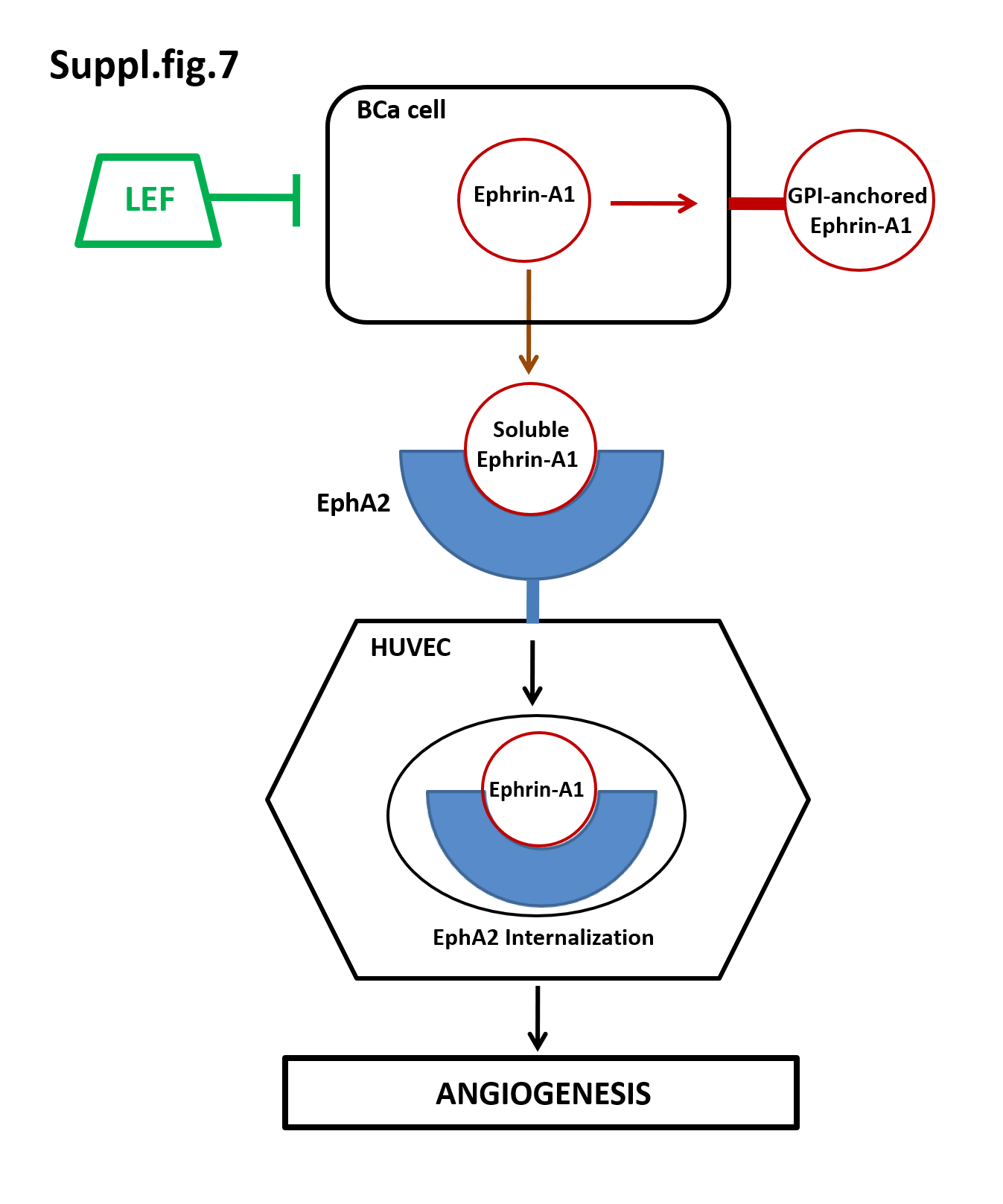


**Supplementary Figure 7. The inhibitory effects of LEF on angiogenesis through suppression of soluble ephrin-A1-mediated activation of its receptor EphA2 on HUVECs (sEphrin-A1/EphA2 system).**

Ephrin-A1 has been characterized as glycosyl phosphatidyl inositol (GPI) - anchored ligand that mediated activation of its receptor EphA2 requiring cell–cell contact. However, in this study, ephrin-A1 was verified to be up-regulated in BCa cells, and another form of ephrin-A1 as soluble functional ligand was found to be released from BCa cells. Soluble ephrin-A1(sEphrin-A1) induced the internalization, subsequently downregulation of EphA2 on HUVECs, then leading to up-regulation of angiogenesis. LEF which is shown in green can effectively suppress the ephrin-A1 expression in BCa cells, subsequently pro-angiogenic function of sEphrin-A1, thus sEphrin-A1/EphA2 system, representing an anti-angiogenesis effect.
